# Motor elements of the third month variously predict individual later motor elements

**DOI:** 10.3389/fnhum.2025.1586228

**Published:** 2025-07-22

**Authors:** Ewa Gajewska, Jerzy Moczko, Magdalena Sobieska

**Affiliations:** ^1^Department of Physiotherapy, Institute of Health Sciences, University of Zielona Gora, Zielona Góra, Poland; ^2^Department of Computer Science and Statistics, Poznan University of Medical Sciences, Poznań, Poland; ^3^Department of Rehabilitation and Physiotherapy, Poznan University of Medical Sciences, Poznań, Poland

**Keywords:** early motor development, axial control, infant neuromotor assessment, preterm infants, motor milestone prediction

## Abstract

**Objective:**

This study aims to determine the correlations between axial and distal motor features observed at 3 months of age on later motor elements at 4–5 and 7–8 months.

**Materials and methods:**

We analyzed 93 children (50 boys); 24 were born prematurely. All children underwent a prospective qualitative evaluation of motor development, performed by the physiotherapist, at 3, 4–5, and 7–8 months of age. We analyzed infants’ motor development in the third month using the validated Quantitative and Qualitative Assessment of Motor Development Worksheet. The qualitative assessment determined for 4–5 and 7–8 months was based on the Vojta development concept.

**Results:**

Studies showed that axial features observed in the third month significantly and relatively strongly correlated (Cramer’s V = 0.4–0.6) with proper asymmetric elbow support. In the supine position, crossing the body’s midline and grasping correlated relatively strongly with the correct positioning of the pelvis and lower limbs observed in the third month. The axial and distal elements from the third month remain necessary to achieve a qualitatively correct oblique sitting position at 7–8 months. The relatively high values of Cramer’s V were also confirmed by the relatively high values of the Tau test. At 7–8 months, this relationship was quite strong sometimes (high Cramer’s V) but applied to a minor part of the variability (low Tau).

**Conclusion:**

Axial elements observed at 3 months of age correlate relatively strongly with axial elements at 4–5 months. The relationships between the elements at 3 months of age and those studied at 7–8 months were moderate but related to axial and distal features. Without proper spine functioning, the development of the shoulders and pelvis will not occur properly. Findings support the early use of axial motor features as developmental indicators for motor progression, with implications for early intervention programs.

## 1 Introduction

Variation is a key characteristic of healthy infant development, and understanding this variation early has important implications for medical intervention and developmental therapy.

Variation is present in practically all developmental parameters, such as motor performance, developmental sequence, or the duration of developmental stages (Touwen, 1976; Hadders-Algra, 2000). The idea that motor behavioral patterns emerge in an orderly genetic sequence resulted in recognizing general developmental rules, such as the cephalocaudal and central-to-distal developmental sequences of development. This thinking became the basis for constructing a structured series of tests to assess developmental milestones (Hadders-Algra, 2000). We have three major theories of infant motor development. Developmental Sequence Theory emphasizes the genetic sequence in motor achievement, such as the cephalocaudal and proximodistal principles (An and Libertus, 2025). Another theory, Dynamic Systems Theory (DST), emphasizes the role of interaction between the individual, task, and environment in organizing spontaneous movement (Thelen and Smith, 1994; Grumi et al., 2022; Smith and Thelen, 2003). DST is flexible but can be too complex for specific predictions. Neuronal Group Selection Theory (NGST) views development as the result of the selection and reinforcement of neural pathways through experience. NGST bridges the genetic and environmental approaches but does not yet explain specific motor sequences in detail (Thelen and Smith, 1994). It is still a matter of debate whether the emergence of more complex movements requires the extinction or integration of primitive reflexes. According to Vojta, the concept of motor development definitely assumes that these primitive reflexes must be extinguished (Dominici et al., 2011).

Our research focuses on another concept, Vojta’s conceptual model, which proposes that development follows a relatively fixed pattern and that perfection in small motor elements at 3 months of age is a prerequisite for complex motor abilities at later ages (Vojta and Peters, 2007).

It emphasizes that while many studies have examined infant motor development, very few have explicitly linked the qualitative performance of 3-month-old infants to specific skills such as asymmetrical elbow support, midline crossing at 4–5 months, and oblique sitting at 7–8 months of age. This gap is important to investigate as it has practical implications in early diagnosis and determining therapeutic interventions (Gajewska et al., 2022).

Our research has been focused on analyzing development after the age of 3 months. The main reason for this is that around 3 months of age, the general movements (GM) are in their final phase and are getting replaced by goal-directed behavior (Hadders-Algra, 2000). Simultaneously, the infant has achieved the ability to balance the head (Silva et al., 2024). Proper head balance is one of the prerequisites for reaching (Hadders-Algra, 2005). Another reason is that 3 months is a period of developmental transition in postural control; the age of major neuro-developmental transition and general movements has considerable predictive power for later developmental disorders (Lisiński et al., 2012; Łojko et al., 2014; Lubkowska and Gajewska, 2020).

Three-month-olds lack the requisite hand control for grasping, but they swat at objects (Needham et al., 2002) and put their hands into their mouths (Van Der Fits, 1999).

Many authors show differences that, at 3 months of age, distinguish between typically developed infants and those with minor neurological dysfunction or CP. These differences include a reduced number of regular movements or postural patterns; the absence of antigravity movements (any movements of legs and/or arms above the level of the trunk); a predominantly flat posture (in supine position all four limbs mainly lying on the surface, antigravity movements and flexion in hips and knees are rare); absence of leg movements toward midline; absence of fiddling movements (Hitzert et al., 2014; Bruggink et al., 2009; Bruggink et al., 2009).

Previous research has shown how individual elements of a qualitative study at 3 months of age affect the performance of selected milestones (observed between 4 and 8 months) and achieving an upright position at 9 months of age (Gajewska et al., 2013; Gajewska et al., 2015; Gajewska et al., 2015; Gajewska et al., 2014; Gajewska et al., 2021). Current research is concentrated on identifying which qualitative motor components observed at 3 months are related to proper elements that compose motor milestones, such as asymmetrical elbow support and midline crossing at 4–5 months and oblique sitting at 7–8 months (Gajewska et al., 2022).

Qualitative assessment regarding the third month of life has been described in many publications (Gajewska et al., 2013; Gajewska et al., 2015; Gajewska et al., 2015; Gajewska et al., 2014; Gajewska et al., 2021; Gajewska and Sobieska, 2015); however, qualitative analysis regarding asymmetrical elbow support and grasping with crossing the center line and oblique sitting has not been presented before. Asymmetrical elbow support is observed between 4 and 5 months of age when the child is lying in a prone position and tries to grasp an object in their field of vision. When attempting target-oriented grasping, the support point is displaced to one elbow. The grasping upper limb is bent at the shoulder joint at an angle of approximately 120 degrees, whereas the spine is set in rotation for the first time (Gajewska et al., 2022; Gajewska et al., 2023). The infant supports themselves on one elbow, the thigh of the lower limb on the same side, and the opposite knee is bent.

Asymmetrical support function is also partially qualitatively described in developmental scales (such as the Alberta Infant Motor Scales) as reaching from forearm support (Eliks et al., 2022; Eliks et al., 2023; Piper et al., 1992; Eliks and Gajewska, 2022), whereas grasping at an oblique angle is described in the Gross Motor Function Measure 88 Scale (Russell et al., 2021; Gajewska et al., 2014).

In the supine position, typically, reaching movements end in grasping around 4 months of age, and the first deliberate grip of the toy appears (Hadders-Algra, 2013).

The function of diagonal grasping, otherwise the first time crossing the body’s midline (grasping and crossing the center line), occurs at 4–5 months (Vojta and Peters, 2007; Fagard et al., 2009). Some authors consider crossing the body’s midline a milestone, which is more frequent with the preferred than with the non-preferred hand (Provine and Westerman, 1979; Savelsbergh et al., 2002).

Around the age of 7–8 months, the grasping upper limb lifts high upward and is bent at an angle of approximately 120 degrees; this body position is called the oblique sit. During this period, the upright trunk is lifted above the ground. This position develops from a safe and solid lateral body position, a prerequisite for pivoting from both the stomach and back positions (Vojta and Peters, 2007; Piper and Darrah, 2022).

### 1.1 Purpose of the study

Identifying which qualitative motor components observed at 3 months relate to proper elements that compose motor milestones such as asymmetrical elbow support, midline crossing at 4–5 months, and oblique sitting at 7–8 months.

## 2 Methods

### 2.1 Participants

The analysis was performed on 93 children (50 boys and 43 girls); 69 children were born on time (gestational week: 39 ± 1), whereas 24 were born prematurely (gestational week: 33 ± 3). The motor development of children born prematurely was analyzed at the corrected age (Hadhud et al., 2023).

Detailed data are given in Table 1.

**Table 1:** Demographic data.

Parameter	Born at term, *n* = 69	Preterm, *n* = 24
Apgar category, 5th minute:		
Severe	1	0
Moderate	0	4
Good	68	20
IVH		
Absent	67	17
I	0	2
II	1	0
III	0	3
IV	1	0
RDS		
Absent	68	18
Present	1	6
Hypotrophy		
Absent	69	22
Present	0	2
Hyperbilirubinemia		
Absent	69	22
Present	0	2
Final motor assessment		
Proper	48	19
Delay	21	5

IVH, Intraventricular Hemorrhage; RDS, respiratory distress syndrome.

Inclusion criteria, recruitment methods: The study group consisted of children born at term or preterm (between week 28 and 42), children reported to the Clinic of Neurology for a periodic assessment of the development with a referral from a general practitioner, a pediatrician or because of parents’ concerns (weak head control in the traction response or suspicion of delayed development). The sample size calculation regarding the number of newborns per year in the area showed the required sample size of 383. However, regarding the number of children with motor disturbances, a sample of 100 was gathered. Not all children appeared for the control testing, so the final number in the sample was 93. The calculation was done using the Statistica.pl. software, assuming the population of the region where the study was performed and the expected percentage of children affected with motor disturbances.

Exclusion criteria encompass genetic or metabolic disorders, severe congenital disabilities, or extreme preterm birth (below the 28th week of gestation). Moreover, no children with microcephaly or macrocephaly were included in the study. Two children born at 28 and 27 weeks of gestation were also recruited, but one did not return for re-evaluation at 7–8 months. That is why we defined children born at <28 weeks of gestation as exclusion criteria. It is also a limitation of our study.

All children underwent prospective evaluation of motor development at 3, 4–5, and 7–8 months of age. The development analysis at 3 months of age was based on the Quantitative and Qualitative Assessment of Motor Development Worksheet, which has already been shown in previous studies (Gajewska et al., 2022; Gajewska et al., 2013; Gajewska et al., 2015; Gajewska et al., 2015; Gajewska et al., 2014; Gajewska et al., 2021; Gajewska and Sobieska, 2015; Gajewska et al., 2023; Gajewska et al., 2021). This type of examination was used in the assessment of children aged 3 months, and the comparison between physiotherapeutic and neurological assessment showed high agreement with high conformity coefficients (z = −5.72483, *p* < 0.001) (Gajewska et al., 2013). The quantitative elements of motor development included asymmetric elbow support and oblique grasp (between 4 and 5 months of age) and oblique sitting (7–8 months of age), which were reported in the paper (Vojta and Peters, 2007; Gajewska et al., 2022).

The qualitative assessment is based on examining small elements of motor development, which is why a binary assessment system (0–1) was introduced. Several scales use multiple-step scoring, but a detailed description is needed to distinguish between the scores. A small element may easily be observed and described as performed in our system.

The group is analyzed as a whole, with no division between children with risk factors and those without them. If the link between the elements exists, it works in typically developing infants and in the presence of disorders. We have shown in previous studies that prematurity itself does not necessarily cause a delay in motor development if children are examined at the corrected age (Gajewska et al., 2022; Gajewska et al., 2021; Gajewska et al., 2018).

### 2.2 Procedure

The qualitative assessment was performed by a physiotherapist (qualified, working in pediatric neurology for 15 years, author of several publications), who assessed three-month-old children in the prone and supine positions (at least 12 weeks completed; the corrected age was considered in the case of preterm babies) (Hadhud et al., 2023), according to the previously described Quantitative and Qualitative Assessment of Motor Development Worksheet (Gajewska et al., 2013; Gajewska et al., 2015; Gajewska et al., 2014; Gajewska et al., 2021; Gajewska and Sobieska, 2015; Gajewska et al., 2023; Gajewska et al., 2021; Gajewska et al., 2018). In the following months, i.e., between 4–5 and 7–8, a qualitative assessment was also performed, as described below. This type of assessment is used by pediatric physiotherapists working with neurodevelopmental methods.

In every case, the physiotherapist conducted the assessment blind without knowing the medical history. In the case of premature children, only the corrected age was known. All clinical data were gathered apart from the testing room.

#### 2.2.1 Qualitative assessment in the third month

**Figure 1:**
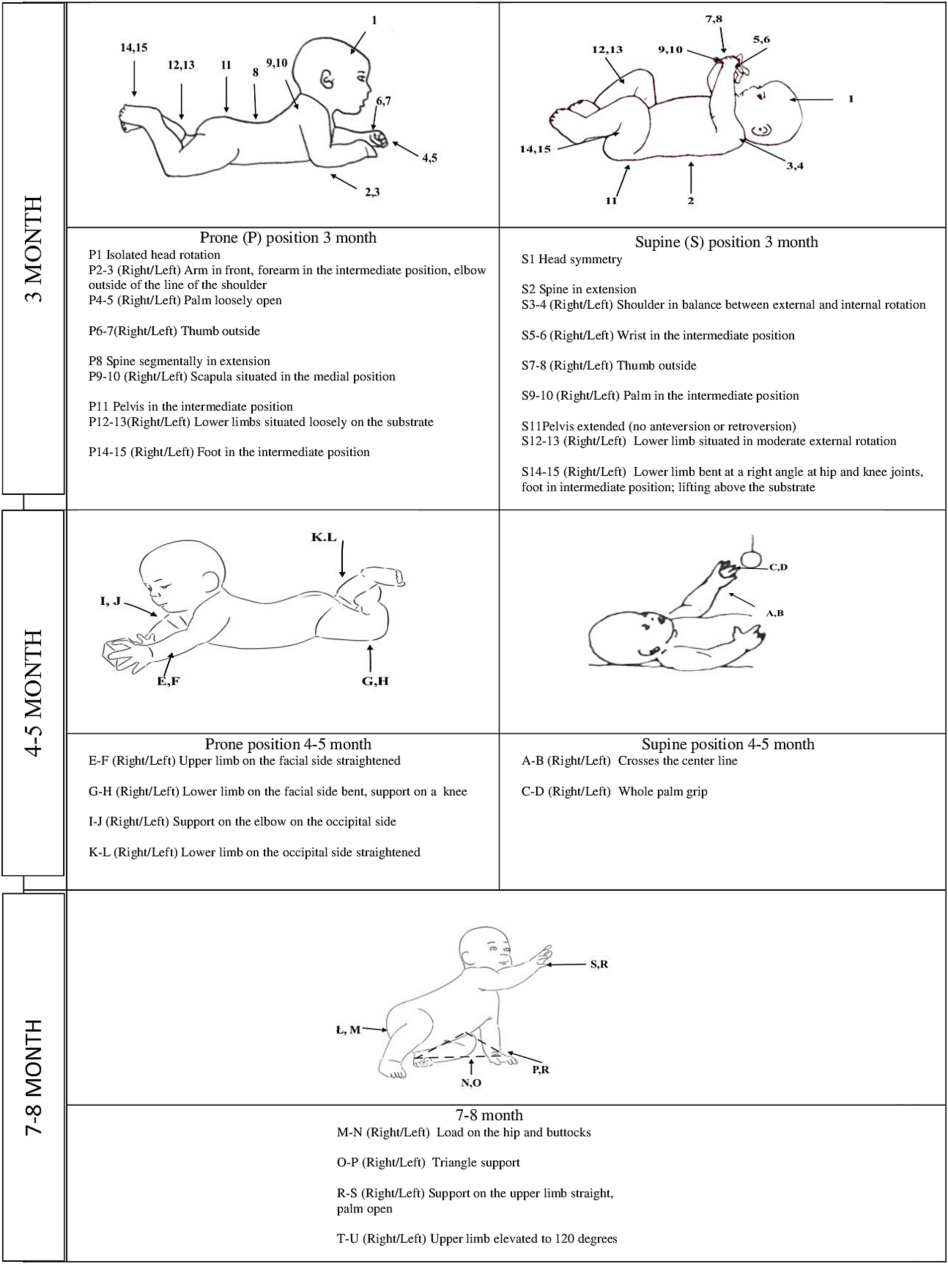
Qualitative assessment of motor development at 3, 4–5, and 7–8 months of life.

A qualitative assessment in the third month included 15 elements, each in the supine and prone positions. In the supine position, the assessment involved head symmetry, spine in extension, shoulder in a balance between external and internal rotation, wrist in an intermediate position, thumb outside, palm in an intermediate position, pelvis extended, lower limb situated in moderate external rotation and bent at the right angle at hip and knee joints, and foot in intermediate position lifting above the substrate (Figure 1). In the prone position, the assessment involved isolated head rotation, arm in front, forearm in an intermediate position, elbow outside of the line of the shoulder, palm loosely open, thumb outside, spine segmentally in extension, scapula situated in medial position, pelvis in an intermediate position, lower limbs situated loosely on the substrate, and foot in an intermediate position (Figure 1) (Gajewska et al., 2013; Gajewska et al., 2015; Gajewska and Sobieska, 2015; Gajewska et al., 2023; Gajewska et al., 2021; Gajewska et al., 2018).

Both sides were assessed for symmetrical parts of the body to exclude asymmetry.

Each element was scored as 0 (element performed only partially or entirely incorrectly) or 1 (element performed entirely correctly). The duration of the examination performed by the physiotherapist was between 10 and 15 min. Each assessed element had to be observed at least 3–4 times during the test. An infant could gather a maximum of 15 points each for the prone and supine positions.

The reliability was first tested with two physiotherapists, assessing 48 children in parallel, and afterward, each of them re-assessed the video recording of 30 children after 1 month. The exact procedure and results are described in the paper. The reliability of the following papers was checked several times, and it was always very high.

Interobserver reliability ranged from 0.870 to 1.000, whereas intra-observer reliability was 1. The comparison between physiotherapeutic and neurological assessment showed high agreement, with high conformity coefficients (z = −5.72483, *p* < 0.001) (Gajewska et al., 2013).

#### 2.2.2 Qualitative assessment at 4–5 months

In the prone position, asymmetrical elbow support was assessed qualitatively by analyzing the following: upper limb on the facial side straightened (right and left side); lower limb on the facial side bent, support on a knee (right and left side); support on the elbow on the occipital side (right and left side), and lower limb on the occipital side straightened (right and left side). In the supine position, the oblique grip was qualitatively assessed by analyzing the following characteristics: crosses the center line to the right and the left and whole palm grip (right and left side) (Figure 1) (Vojta and Peters, 2007; Gajewska et al., 2022; Piper and Darrah, 2022).

#### 2.2.3 Qualitative assessment at 7–8 months

A qualitative assessment in the 7–8 months included: load on the hip and buttocks (right and left side); triangle support (right and left side); support on the upper limb straight, palm open (right and left side); upper limb elevated to 120 degrees (right and left side) (Figure 1) (Vojta and Peters, 2007; Piper and Darrah, 2022).

Each element of the qualitative developmental assessment at 4–5 and 7–8 months was assessed as 0 (element performed only partially or entirely incorrectly) or 1 (element performed entirely correctly). Each assessed element had to be observed at least 3–4 times during the same assessment.

### 2.3 Ethics declarations

The examination was performed at the Center for Child and Adolescent Neurology and the Child Clinic from 2016 to 2019. Informed consent was obtained from all of the participant’s parents or caregivers, and the study was approved by the Local University Research Ethics Committee [registered under no. 22/10 (07-01-2010)]. The study conformed to all ethical issues included in the Declaration of Helsinki.

### 2.4 Statistics analyses

The mean and standard deviation were used to describe interval variables after a preliminary assessment of the normality of the distribution with the Shapiro–Wilk test. The analysis involved examining the relationship each time between the two dichotomous variables [the child performs the item or not (0/1)] using Cramer’s test (the V coefficient allowed to assess the strength of the relationship between the variables) and Kendall’s Tau, which shows how much of the variability in one variable is accounted for by the other, with Bonferroni correction. In both cases, a higher coefficient value indicated greater relationship strength. A *p*-value of <0.05 showed the significance of these relationships.

Based on a suggestion from Lee, the values of Cramer’s V should be interpreted as follows: 0.00–0.10 negligible, 0.10–0.20 weak, 0.20–0.40 moderate, 0.40–0.60 relatively strong, 0.60–0.80 strong and 0.80–1.00 very strong (Lee, 2016).

## 3 Results

The positions and the elements assessed are presented in Figure 1. For more clarity, the results are presented as a summary in the paper (Tables 2, 3). In contrast, the supplementary material includes exact values depicting the relationships’ strength (Supplementary Tables I–IV).

**Table 2:** Elements assessed in the prone position in the 3rd, prone and supine position in the 4–5 and 7–8 months.

Elements assessed in third month, in the prone position	Elements assessed in 4–5 months, in the prone position	Elements assessed in 7–8 months, in the prone position
P1: Isolated head rotation P2–3: Arm in front, forearm in the intermediate position, elbow outside of the line of the shoulder, right and left P4–5: Palm loosely open, right and left P6–7: Thumb outside, right and left P8: Spine segmentally in extension P9–10: Scapula situated in the medial position, right and left P11: Pelvis in the intermediate position P12–13: Lower limb situated loosely on the substrate, right and left P14–15: Foot in the intermediate position, right and left	Upper limb on the facial side straightened—right and left Correlates with: P1, P2–3, P4–5, P6–7, P8, P9–10, P11, P12–13 Lower limb on the facial side bent; support on a knee—right and left Correlates with: P1, **P2–3**, P4–5, P6–7, P8, P9–10, P11, P12–13 Supports on the elbow on the occipital side—right and left Correlates with: P1, P2–3, P4–5, P6–7, P8, P9–10, P11, P12–13 Lower limb on the occipital side straightened—right and left Correlates with: P12–13, P11, P14–15	Load on the hip and buttocks, right and left Correlates with: P1 Triangle support, right and left Correlates with: P2–3, P11 Support on the upper limb straight, palm open, right and left Correlates with: P4–5, P6–7, P14–15 Upper limb elevated to 120 degrees, right and left Correlates with: P1, P2–3, P4–5, P6–7, P11, P12–13
	The supine position	
	Crosses the center line to the right or left Correlates with: P1, P2–3, P4–5, P6–7, P8, P9–10, P 11, P12–13 Whole palm grip—right and left Correlates with: P1, P2–3, P4–5, P6–7, P8, P9–10, P11, P12–13	

P, prone. All the relatively strong (Cramer’s V = 0.40–0.60) correlations are listed, one strong correlation (Cramer’s V = 0.60–0.80) is marked in bold; exact values are given in the Supplementary Tables I–IV.

**Table 3:** Elements assessed in the supine position in the 3rd, in the supine and prone position in the 4–5 months and prone position in the 7–8 months.

Elements assessed in third month, in the supine position	Elements assessed in 4–5 months, in the supine position	Elements assessed in 7–8 months, in the prone position
S1: Head symmetry S2. Spine in extension S3–4: Shoulder in balance between external and internal rotation, right and left S5–6: Wrist in intermediate position, right and left S7–8: Thumb outside, right and left S9–10: Palm in the intermediate position, right and left S11: Pelvis extended (no anteversion or retroversion) S12–13: Lower limb situated in moderate external rotation, right and left S14–15: Lower limb bent at a right angle at hip and knee, joints, foot in intermediate position; lifting above the substrate, right and left	Crosses the center line to the right or left Correlates with S2, S3–4, S5–6, S11, S12–13 Whole palm grip—right and left Correlates with: S2, S3–4, S5–6, S9–10, S11, S12–13, S14–15	Load on the hip and buttocks right and left Correlates with: S2 Triangle support right and left Correlates with: S2, S3–4, S11 Support on the upper limb straight, palm open right and left Correlates with: S5–6, S7–8, S9–10 Upper limb elevated to 120 degrees right and left Correlates with: S1, S2, S3–4, S5–6, S7–8, S9–10, S11
	In the prone position	
	Upper limb on the facial side straightened—right and left Correlates with: S2, S3–4, S5–6, S7–8, S9–10, S11, S12–13, S14–15 Lower limb on the facial side bent; support on a knee—right and left Correlates with: S1, S2, S3–4, S5–6, S7–8, S9–10, S11, S12–13, S14–15 Supports on the elbow on the occipital side—right and left Correlates with: S1, S2, S3–4, S5–6, S7–8, S9–10, S11, S12–13, S14–15 Lower limb on the occipital side straightened—right and left Correlates with: S3–4, S11, S12–13	

S, supine. All the relatively strong (Cramer’s V = 0.40–0.60) correlations are listed; exact values are given in the Supplementary Tables I–IV.

With a few exceptions, all the relationships between the variables studied were statistically significant (*p* < 0.05). Given the value of Cramer’s V coefficient, powerful relationships could be identified. They tended to appear on the same side of the body. For ease of interpretation, Tables 2, 3 highlight the existence of relatively strong correlations, regardless of whether the highest values were on both or one side of the body. Detailed data are given in the tables in the supplementary material. For each pair of correlations, the values of the statistics are given in the following order: the value of Cramer’s V coefficient, the confidence range of this coefficient, the value of Goodman-Kendal’s Tau coefficient, and the *p*-value. The relatively strong Cramer’s V coefficient values (0.4–0.6) are marked with yellow, and one value that exceeded 0.6 is indicated in bold.

The correlation between the elements assessed in the third month and those assessed in 4–5 months mainly was relatively strong or moderate. The correlation between the elements assessed in the third month and those assessed in 7–8 months was mostly moderate, but some relatively strong correlations were present.

The data in Tables 2, 3 show a strong relationship between axial features assessed in the third month (head, shoulders, spine, pelvis) and almost all elements assessed in the 4–5 months, both in the prone and supine positions. The correlations between assessment in the same position (prone or supine) are as strong as those between adverse positions (prone and supine and vice versa). The distal elements assessed in the third month (wrist, palm, thumb, foot) are moderately correlated with most of the elements assessed in the 4–5 months. The correlations were usually present for the same side of the body (an element assessed on the left side in the third month correlated with an element assessed on the left side in the 4-to 5 months). However, sometimes, the strength of the correlation differed and dropped to moderate values.

The relationship between variables assessed in the third month and those assessed in 7–8 months showed the pelvis’s relatively strong relation, both in the prone and the supine position with “Triangle support” and “Upper limb elevated to 120 degrees.” Less relatively strong correlations were observed for the position of the spine and shoulders. Other relatively strong relations were observed between distal elements of the upper limb (wrist, palm, thumb) assessed in the third month with “Support on the upper limb straight” and “Upper limb elevated to 120 degrees.”

The relatively high values of Cramer’s V were also confirmed by the relatively high values of the Tau test; that is, elements from the third month accounted for a significant part of the variability of the elements studied at 4–5 months of age. At 7–8 months, this relationship was quite strong sometimes (high Cramer’s V) but applied to a minor part of the variability (low Tau).

Correctly performed arm in front, forearm in the intermediate position, and elbow outside of the shoulder line (third month) showed a strong relationship with elbow support on the right and left sides between 4 and 5 months. At the same time, Kendall’s Tau coefficient values showed that the relationship between the upper limb motility at 3 months accounted for approximately 30% of the variation in the motility of the same limb between months four and five. A correlation was observed between correct elbow support at 3 months and lower limb motility for months four and five, both flexed and upright on the correct side.

In the supine position, grasping with crossing the center line—mainly a function of the upper limbs—was assessed at 4–5 months of age (Table 2; Figure 1; Supplementary Table II).

When the head position (symmetry) and rotation were assessed, the rotation feature mainly correlated with elements assessed at 4–5 months. In the 7–8 month, “Load on the hip and buttock” and “Upper limb elevated to 120 degrees” correlated relatively strongly with head rotation. Both elements correlated relatively strongly with spine extension assessed in the supine position in the third month and only moderately with spine segmentally in extension, as assessed in the prone position.

## 4 Discussion

The achievement of milestones such as sitting, crawling, and walking in the first years of life reflects proper motor development: most assessment systems include their observation, but physiotherapists consider limiting themselves to this assessment as an insufficient way of examining motor performance. Observing achievement at critical points does not reflect the complex process of motor development. Moreover, milestones that evolve in the first year of life with excessive variation (Dosman et al., 2012) do not explain the delay in their achievement. Research on motor development addresses this gap (Berger et al., 2021; Law and Darrah, 2014). Some studies point to the value of laboratory findings, whereas others are limited to observing natural progress in motor development (Sylos-Labini et al., 2020; De Bartolo et al., 2022). A more robust assessment shown in the literature involves observing a child’s progressively acquired motor skills in detail, which are both the basis and catalyst for development, including perception, cognitive development, and social interaction (Ferronato et al., 2021).

Another approach to motor development is to assess its quality, describing not just the movement itself but the way it is performed, which can allow earlier detection of motor disorders in children and thus avoid the loss of valuable information and intervention time that can be associated with waiting to reach critical milestones (Johansen et al., 2017). Hence, this paper (as another by this team of authors) concerns qualitative assessment, i.e., a detailed study of small elements that make up large motor functions.

This approach has been promoted in studies by Gajewska et al. (2013, 2014, 2018) and Johansen et al. (2017). Severe and the most minor motor impairments can be detected by assessing the quality of function performance. Qualitative assessment’s effect on achieving milestones such as turning, sitting, crawling, or standing up has been described previously. Previously, we conducted the same type of analysis of the elements assessed in the third month and the sixth month of life (Gajewska et al., 2021). The analysis used the third month of life as described and shown in the Quantitative and Qualitative Assessment of Motor Development Worksheet, as well as developmental features between the fourth and fifth (asymmetrical elbow support and oblique grasp) and between the seventh and eighth months (oblique sit).

The influence of achieving correct motor function in the third month of life on achieving the corresponding functions in months 4–5 and 7–8 has been described in Gajewska et al. (2022). This article focuses on the correlation between individual motor elements from the third month and the elements that constitute the corresponding functions in later development.

The association between the variables, showing high values of Cramer’s V, indicates the existence of a relationship that is not merely random between the performance of a motor element in the third month and the appearance of a particular expected element between the 4th and 5th and seventh and eighth months, respectively. The relationship is based on practical observations describing the process of motor development and demonstrated mathematically. All observed correlations were statistically significant but differed in strength, from weak (Cramer’s V 0.10–0.20) to strong (0.60–0.80) in very few cases (Palm loosely open in the third month with Whole palm grip in 4–5 months).

The axial features in the prone position in the third month (arm in front, scapulas, spine in extension, pelvis, and lower limbs situated loosely on the substrate) all show relatively strong correlations with all elements of the 4–5 months, both in prone and supine position. The relationship between distal elements (wrists, palms, thumbs) is still significant but moderately strong. The same axial elements from the third month correlate less strongly with elements observed in 7–8 months; only the position of the pelvis remains crucial for “upper limb elevated to 120 degrees.” However, the distal elements of the upper limb correlate more strongly with elements observed in 7–8 months. It is worth noting that these were conditioned (in addition to the apparent influence of shoulder position at 3 months) by the correct pelvis and lower limbs’ positioning. The axial features were still decisive for the correct occurrence of distal features.

It is also worth noting that the critical skill of crossing the line of the center of the body and reaching beyond it (oblique grasp) appears in the period between the assessment (that is, 3 months and the next stage, 4–5 months) (Van Der Fits, 1999; Gajewska et al., 2014; Gajewska et al., 2021; Gajewska and Sobieska, 2015). These skills only appear if an infant achieves symmetrical support on both elbows and proper spine and pelvis alignment at 3 months. It was proved in an earlier paper (Gajewska et al., 2022). By 3 months, achieving balance in complete symmetry seems crucial. From 4 to 5 months onward, crossing the line of the body’s center (diagonal body positioning) appears as a prelude to alternate movement and, eventually, crawling and alternate bipedal gait (Vojta and Peters, 2007). Hence, observing asymmetrical diagonal support and grasping is essential for further harmonious development. The grasping with the crossing the line of the center of the body requires the action of a shoulder girdle, but the position of the pelvis guarantees good balance. We observed a relatively strong correlation between the position of the pelvis and asymmetric features observed in 4–5 months. The next stage is the gradual elevation of the upper torso above the ground, assessed as an oblique sit at 7–8 months. We saw a strong correlation between the position of the pelvis in the third month and the “Upper limb elevated to 120 degrees” in 7–8 months.

Proper spinal alignment, assessed in the supine position, does not guarantee the achievement of subsequent skills at the following time points tested. In contrast, when assessed in the prone position, which requires antigravity action, it strongly predicts subsequent skills.

This consequence becomes even more pronounced when the relationships between items assessed in the third month of life and later ones not in the same position, but vice versa, were analyzed: items assessed in the prone position are stronger predictors of the occurrence of corresponding skills also in the supine position than vice versa.

Other correlations were weak. According to Vojta’s concept, isolated head rotation and its symmetrical position are the final functions described for the movement of this part of the spine. Achieving this feature in the third month is necessary for further development but does not change later. Cranio-caudal development ends in the third month, and proximal-distal development appears (Vojta and Peters, 2007; Hadders-Algra, 2005). Our data confirm this theory.

This study can improve motor assessment methods or early intervention strategies. The approach to motor development as successive milestones is insufficient. The assessment method presented in the article does not only capture delays in motor development or severe movement disorders. It focuses on significant or minor abnormalities such as asymmetry of development or lack of proper posture. It answers why an infant does not perform a given motor feature (at all or on time) if he/she did not achieve proper motor performance at 3 months.

Understanding normal motor development can help clinicians recognize delays early and provide children with adequate early intervention, which may help them achieve key developmental milestones. Studies on early intervention outcomes have shown various benefits when children receive the necessary speech therapy, physiotherapy, and occupational therapy.

The authors know that the study group is not very large and includes relatively many infants born preterm but not extremely preterm. Like all developmental scales, the assessment remains subjective and has not yet been validated against other motor developmental scales apart from the Denver test and neurological assessment (Gajewska et al., 2013; Gajewska and Sobieska, 2015).

## 5 Conclusion

Axial elements observed at 3 months of age correlate relatively strongly with axial elements at 4–5 months. The relationships between the elements at 3 months of age and those studied at 7–8 months of age are moderate but relate to both axial and distal features. Without proper spine functioning, the development of the shoulders and pelvis will not occur properly.

Findings support the early use of axial motor features as developmental indicators for motor progression, with implications for early intervention programs.

### 5.1 Limitations of the study

The study is limited to infants born after 28 weeks of gestation. Future studies conducted on a larger group should include observation by more than one physiotherapist.

## Data Availability

The original contributions presented in the study are included in the article/Supplementary material, further inquiries can be directed to the corresponding author.
