# Supplementary material for: Motor elements of the third month variously predict individual later motor elements
Source: Front Hum Neurosci. 2025 Jul 22;19:1586228. doi: 10.3389/fnhum.2025.1586228 (PMC12322975; doi:10.3389/fnhum.2025.1586228)
Supplement: Supplementary file 1 [file Supplementary_file_1.zip › Supplementary Material/Table_1.DOCX]

Table I. The impact of qualitative elements assessed at three months of age in the prone position on qualitative elements assessed at the age of 4–5 months in the prone and supine position. For each pair of variables, the values of Cramer’s V coefficient, confidence intervals, and Goodman and Kruskal Tau coefficients are given, along with the exact p-value. The relatively strong correlations are marked with yellow, the strong correlation is marked in bold.

| Qualitative characteristics in the prone position, 3^rd^ month | Side of the body | 4-5 month, the prone position | | | | | | | | 4-5 month, the supine position | | | |
| --- | --- | --- | --- | --- | --- | --- | --- | --- | --- | --- | --- | --- | --- |
|  |  | Upper limb on the facial side straightened - R | Upper limb on the facial side straightened - L | Lower limb on the facial side bent; support on a knee - R | Lower limb on the facial side bent; support on a knee L | Supports on the elbow on the occipital side - R | Supports on the elbow on the occipital side - L | Lower limb on the occipital side straightened - R | Lower limb on the occipital side straightened - L | Crosses the center line to the R | Crosses the center line to the L | Whole palm grip  R | Whole  palm grip  L |
| Isolated head rotation, Y/N | - | 0.4148 (0.2068–0.6227); 0.1720;  0.0001 | 0.4330 (0.2265–0.6394); 0.1875;  0.0001 | 0,4724 (0.2787–0.6741); 0.2332;  0.0000 | 0.4616 (02596–0.6336); 0.2131;  0.0000 | 0.4661 (0.2637–0.6686); 0.2173;  0.0000 | 0.5244 (0.3333–0.7155); 0.2750;  0.0000 | 0.3932 (0.1831–0.6033); 0.1576;  0.0004 | 0.3570 (0.1431–0.5709); 0.1274;  0.0013 | 0.4330 (0.2265-0.6394);  0.0001; 0.1920 | 0.3686 (0.1583-0.5790); 0.0007; 0.1480 | 0.4330 (0.2265-0.6394);  0.0001; 0.1920 | 0.3570 (0.1431-0.5709); 0.0015; 0.1261 |
| Arm in front, forearm in the intermediate position, elbow outside of the line of the shoulder, Y/N | Right | 0.5708 (0.3706–0.7710); 0.3258;  0.0000 | 0.3914 (0.1671–0.6157); 0.3999;  0.0005 | **0.6324 (0.4432–0.8216); 0.1736;**  **0.0000** | 0.4167 (0.1932–0.6401); 0.3134;  0.0002 | 0.5599 (0.3561–0.7636); 0.1736;  0.0000 | 0.4167 (0.1932–0.6401); 0.1620;  0.0002 | (0.1760–0.6289); 0.1291;  0.0004 | 0.3593 (0.1286–0.5901); 0.2007;  0.0016 | 0.4868 (0.2384-0.6753); 0.0001; 0.2087 | 0.5237 (0.3196-0.7278); 0.0001; 0.2742 | 0.5878 (0.3886-0.7870); 0.0000; 0.3465 | 0.4323 (0.2087-0.6559); 0.0002; 0.1869 |
|  | Left | 0.3672 (0.1475–0.5869); 0.1348;  0.0008 | 0.4382 (0.2207–0.6557); 0.1920;  0.0001 | 0.4279 (0.2125–0.6433); 0.1831;  0.0001 | 0.5311 (0.3256–0.7366); 0.2821;  0.0000 | 0.4762 (0.2636–0.6887); 0.2267;  0.0000 | 0.5311 (0.3256–0.7366); 0.2821;  0.0000 | 0.3162 (0.0871–0.5454); 0.1000;  0.0043 | 0.3439 (0.1154–0.5724); 0.1920;  0.0023 | 0.4382 (0.2207-0.6557); 0.0001; 0.1928 | 0.3847 (0.1687-0.6086); 0.0005; 0.1480 | 0.4382 (0.2207-0.6557); 0.0001; 0.1920 | 0.3847 (0.1687-0.6086); 0.0005; 0.1729 |
| Palm loosely open, Y/N | Right | 0.5861 (0.4128–0.7594); 0.3435;  0.0000 | 0.3510 (0.0917–0.6103); 0.1232;  0.0043 | 0.4928 (0.2814–0.7042); 0.2428;  0.0001 | 0.3682 (0.1041–0.6323); 0.1356;  0.0031 | 0.5292 (0.3115–0.7468); 0.2800;  0.0000 | 0.3682 (0.1041–0.6323); 0.1356;  0.0031 | 0.2998 (0.0217–0.5779); 0.0899;  0.0148 | 0.3187 (0.0342–0.6032); 0.1016;  0.0111 | 0.4070 (0.2132-0.6009); 0.0004;  0.1232 | 0.5511 (0.3831-0.7192); 0.0000; 0.3037 | 0.6487 (0.4688-0.8285); 0.0000; 0.4208 | 0.4293 (0.1499-0.7087); 0.0011; 0.1843 |
|  | Left | 0.4459 (0.2272–0.6646); 0.1988;  0.0003 | 0.3923 (0.1376–0.6471); 0.1539;  0.0019 | 0.4459 (0.2272–0.6646); 0.1988;  0.0003 | 0.4899 (0.1503–0.6695); 0.1680;  0.0013 | 0.4792 (0.2530–0.7054); 0.2296;  0.0001 | 0.4899 (0.1503–0.6695); 0.1680;  0.0013 | 0.3358 (0.0542–0.6174); 0.1128;  0.0004 | 0.3551  (00670–0.6433); 0.1261;  0.0062 | 0.3923 (0.1376-0.6471); 0.0020; 0.1539 | 0.3207 (0.0868-0.5547); 0.0075; 0.1029 | 0.4978 (0.2677-0.7279); 0.0000; 0.2478 | 0.4727 (0.1976-0.7477); 0.0004; 0.2234 |
| Thumb outside, Y/N | Right | 0.4070 (0.2132–0.6009); 0.2428;  0.0004 | 0.2518 (0.0095–0.5131); 0.0634;  0.0300 | 0.4928 (0.2814–0.7043); 0.2428;  0.0001 | 0.3682 (0.1041–0.6323); 0.1356;  0.0031 | 0.4321 (0.1900–0.6742); 0.1867;  0.0005 | 0.2666 (0.0474–0.5331); 0.0711;  0.0242 | 0.2998 (0.0217–0.5779); 0.0899;  0.0148 | 0.3187 (0.0342–0.6032); 0.1016;  0.0111 | 0.2518 (0.0095-0.5131); 0.0346; 0.0634 | 0.4609 (0.2556-0.6662); 0.0001; 0.2124 | 0.5494 (0.3288-0.7701); 0.0001; 0.3019 | 0.3178 (0.0342-0.6032); 0.0122; 0.1016 |
|  | Left | 0.3467 (0.1051–0.5883); 0.1202;  0.0043 | 0.2869 (0.0227–0.5511); 0.0823;  0.0171 | 0.4459 (0.2272–0.6646); 0.1988;  0.0003 | 0.4899 (0.1503–0.6695); 0.1680;  0.0013 | 0.3760 (0.1259–0.6262); 0.1414;  0.0025 | 0.3819 (0.0323–0.5715); 0.0911;  0.0141 | 0.3358 (0.0542–0.6174); 0.1128;  0.0004 | 0.3551 (0.0670–0.6433); 0.1261;  0.0062 | 0.2869 (0.0227-0.5511); 0.0196; 0.0823 | 0.2248 (0.0172-0.4669); 0.0516; 0.0505 | 0.3923 (0.1376-0.6471); 0.0020; 0.1539 | 0.3551 (0.0670-0.6433); 0.0066; 0.1261 |
| Spine segmentally  in extension, Y/N | - | 0.4386 (0.2378–0.6395); 0.1924;  0.0001 | 0.4624 (0.2663–0.6585); 0.2138;  0.0000 | 0.5516 (0.3673–0.7359); 0.3843;  0.0000 | 0.4927 (0.3034–0.6828); 0.2427;  0.0000 | 0.5515 (0.3701–0.7330); 0.3842;  0.0000 | 0.5542 (0.3784–0.7299); 0.3071;  0.0000 | 0.3657 (0.1588–0.5726); 0.1338;  0.0009 | 0.3311 (0.1288–0.5414); 0.1096;  0.0025 | 0.3423 (0.1319-0.5526); 0.0018; 0.1171 | 0.4992 (0.3058-0.6926); 0.0000; 0.2492 | 0.4624 (0.2663-0.6585); 0.0000; 0.2138 | 0.3980 (0.1973-0.5987); 0.0003; 0.1584 |
| Scapula situated i  n the medial position, Y/N | Right | 0.5137 (0.2993–0.7281); 0.2639;  0.0000 | 0.3814 (0.1487–0.6141); 0.1454;  0.0008 | 0.5786 (0.3737–0.7835); 0.3248;  0.0000 | 0.4850 (0.1718–0.6382); 0.1640;  0.0004 | 0.4943 (0.2733–0.7153); 0.2443;  0.0000 | 0.4050 (0.1718–0.6382); 0.1640;  0.0004 | 0.3883 (0.1450–0.6216); 0.1469;  0.0009 | 0.3341 (0.0918–0.5764); 0.1116;  0.0036 | 0.3124 (0.0774-0.5473); 0.0058; 0.0976 | 0.4710 (0.2563-0.6857); 0.0000; 0.2218 | 0.5194 (0.2996-0.7391); 0.0000; 0.2697 | 0.4110 (0.1731-0.6489); 0.0005; 0.1689 |
|  | Left | 0.3245 (0.1010–0.5479); 0.1053;  0.0028 | 0.3914 (0.1671–0.6157); 0.1532;  0.0005 | 0.3861 (0.1655–0.6066); 0.1490;  0.0005 | 0.4837 (0.2676–0.6998); 0.2340;  0.0000 | 0.4837 (0.2676–0.6998); 0.1864;  0.0000 | 0.4837 (0.2676–0.6998); 0.2340;  0.0000 | 0.2610 (0.0282–0.4938); 0.0681;  0.0153 | 0.2864 (0.0524–0.5204); 0.0820;  0.0094 | 0.3259 (0.0982-0.5536); 0.0036; 0.1062 | 0.2855 (0.0641-0.5070); 0.0096; 0.0815 | 0.3914 (0.1671-0.6157); 0.0006; 0.1532 | 0.3593 (0.1286-0.5901); 0.0018; 0.1291 |
| Pelvis in the  intermediate position, Y/N | - | 0.3861 (0.1655–0.6066); 0.1490;  0.0005 | 0.5223 (0.3121–0.7325); 0.2728;  0.0000 | 0.5708 (0.3706–0.7710); 0.3258;  0.0000 | 0.4837 (0.2676–0.6998); 0.2340;  0.0000 | 0.5599 (0.3561–0.7636); 0.3134;  0.0000 | 0.5708 (0.3706–0.7710); 0.3033;  0.0000 | 0.4732 (0.2551–0.6913); 0.2239;  0.0000 | 0.4323 (0.0287–0.6559); 0.1869;  0.0002 | 0.4568 (0.2384-0.6753); 0.0001; 0.2087 | 0.4641 (0.2531-0.6752); 0.0000; 0.2154 | 0.5223 (0.3121-0.7325); 0.0000; 0.2728 | 0.4323 (0.2087-0.6559); 0.0002; 0.1869 |
| Lower limbs situated loosely on the substrate, Y/N | Right | 0.5011 (0.2789–0.7234); 0.2511;  0.0000 | 0.4819 (0.2415–0.7223); 0.2322;  0.0001 | 0.5792 (3735–0.7849); 0.3355;  0.0000 | 0.5894 (0.3622–0.8167); 0.3474;  0.0000 | 0.5421 (0.3166–0.7676); 0.2939;  0.0000 | 0.5044 (0.2621–0.7467); 0.2544;  0.0000 | 0.5555 (0.3110–0.8000); 0.3086;  0.0000 | 0.5848 (0.3407–0.8294); 0.3422;  0.0000 | 0.4819 (0.2415-0.7223); 0.0001; 0.2322 | 0.4650 (0.2467-0.6834); 0.0001; 0.2163 | 0.4819 (0.2415-0.7223); 0.0001; 0.2322 | 0.4923 (0.2336-0.7511); 0.0001; 0.2424 |
|  | Left | 0.4683 (0.2409–0.6957); 0.2139;  0.0001 | 0.4512 (0.2089–0.6935); 0.2036;  0.0002 | 0.5437 90.3287–0.7587); 0.2956;  0.0000 | 0.5557 (0.3232–0.7881); 0.3088;  0.0000 | 0.5089 (0.2783–0.7395); 0.2590;  0.0000 | 0.4735 (0.2294–0.7177); 0.2242; 0.0001 | 0.5240 (0.2776–0.7704); 0.2746;  0.0000 | 0.5529 (0.3068–0.7990); 0.3057;  0.0000 | 0.4512 (0.2089-0.6935); 0.0002; 0.2036 | 0.3593 (0.1286-0.5901); 0.0018- 0.1291 | 0.4512 (0.2089-0.6935); 0.0002; 0.2036 | 0.3740 (0.1105-0.6376); 0.0023;  0.1389 |
| Foot in the  intermediate position, Y/N | Right | 0.3467 (0.1051–0.5883); 0.1202;  0.0043 | 0.2869 (0.0227–0.5511); 0.0823;  0.0177 | 0.4459 (0.2272–0.6646); 0.1988;  0.0003 | 0.4099 (01503–0.6695); 0.1680;  0.0013 | 0.3760 (0.1259–0.6262); 0.1414;  0.0025 | 0.3019 (0.0323–0.5715); 0.0911;  0.0141 | 0.3358 (0.0542–0.6174); 0.1128;  0.0084 | 0.3551 (0.0670–0.6433); 0.1261;  0.0062 | 0.2869 (0.0227-0.5511); 0.0196; 0.0823 | 0.3207 (0.0868-0.5547); 0.0076; 0.1029 | 0.3923 (0.1376-0.6471); 0.0020; 0.1539 | 0.3551 (0.0670-0.6433); 0.0066; 0.1261 |
|  | Left | 0.3467 (0.1051–0.5883); 0.1202;  0.0043 | 0.2869 (0.0227–0.5511); 0.0823;  0.0177 | 0.4459 (0.2272–0.6646); 0.1988;  0.0003 | 0.4099 (01503–0.6695); 0.1680;  0.0013 | 0.3760 (0.1259–0.6262); 0.1414;  0.0025 | 0.3358 (0.0542–0.6174); 0.0911;  0.0084 | 0.3358 (0.0542–0.6174); 0.1128;  0.0084 | 0.3551 (0.0670–0.6433); 0.1261;  0.0062 | 0.2869 (0.0227-0.5511); 0.0196; 0.0823 | 0.2248 (0.0172-0.4669); 0.0516; 0.0505 | 0.3923 (0.1376-0.6471); 0.0020; 0.1539 | 0.2376 (0.0476-0.5218); 0.0544; 0.0565 |
