# Supplementary material for: Motor elements of the third month variously predict individual later motor elements
Source: Front Hum Neurosci. 2025 Jul 22;19:1586228. doi: 10.3389/fnhum.2025.1586228 (PMC12322975; doi:10.3389/fnhum.2025.1586228)
Supplement: Supplementary file 1 [file Supplementary_file_1.zip › Supplementary Material/Table_2.DOCX]

Table II. The impact of qualitative elements assessed at three months of age in the supine position on qualitative elements assessed at the age of 4–5 months in the prone and supine position. For each pair of variables, the values of Cramer’s V coefficient, confidence intervals, and Goodman and Kruskal Tau coefficients are given, along with exact p-value. The relatively strong correlations are marked with yellow.

| Qualitative characteristics in the supine  position | Side of the body | 4-5 month, the prone position | | | | | | | | 4-5 month, the supine position | | | |
| --- | --- | --- | --- | --- | --- | --- | --- | --- | --- | --- | --- | --- | --- |
|  |  | Upper limb on the facial side straightened - R | Upper limb on the facial side straightened - L | Lower limb on the facial side bent; support on a knee - R | Lower limb on the facial side bent; support on a knee L | Supports on the elbow on the occipital side - R | Supports on the elbow on the occipital side - L | Lower limb on the occipital side straightened - R | Lower limb on the occipital  side  straightened - L | Crosses the center line to the R | Crosses the center line to the L | Whole palm grip R | Whole palm grip L |
| Head symmetry Y/N | - | 0.3630  (0.1374-0.5886); 0.0012;  0.1318 | 0.3619  (0.1307-  0.5730);  0.0015;  0.1309 | 0.4267 (0.2052-0.6482); 0.0002; 0.1821 | 0.3855 (0.1559-0.6171); 0.0000; 0.1486 | 0.4548 (0.2289-0.6808); 0.0001; 0.1648 | 0.3651 (0.1292-0.6011); 0.0016; 0.2069 | 0.3173 (0.0778-0.5568); 0.0059;  0.1333 | 0.2942  (0.0613-  0.5270);  0.0085;  0.1007 | 0.2942 (0.0613-0.5270); 0.0865;  0.0071 | 0.2629 (0.0385-0.4873); 0.0691;  0.0120 | 0.3619 (0.1307-0.5930); 0.1309;  0.0013 | 0.2418 (0.0026–0.4810); 0.0585;  0.0243 |
| Spine in extension, Y/N | - | 0.4552  (0.2539-0.06565); 0.0000;  0.2072 | 0.4174  (0.2120-  0.6228);  0.0002;  0.1742 | 0.5693 (0.3851-0.7535); 0.0000; 0.3241 | 0.4461 (0.2452-0.6470); 0.0001; 0.1990 | 0.5684 (0.3867-0.7502); 0.0000;  0.3231 | 0.5082 (0.3180-0.6984); 0.0000; 0.2583 | 0.3792 (0.1707-0.5877); 0.0007;  0.1438 | 0.3438  (0.1318-  0.5559);  0.0020;  0.1182 | 0.3567 (0.1451-0.5683); 0.1272;  0.0010 | 0.4620 (0.2619-0.6622); 0.2135;  0.0000 | 0.4788 (0.2811-0.6750); 0.2285;  0.0000 | 0.3438 (0.1318–0.5559); 0.1182;  0.0018 |
| Shoulder in balance between external and internal rotation, Y/N | Right | 0.4722  (0.2513-0.6932); 0.0000;  0.2230 | 0.4021  (0.1680-  0.6361);  0.0005;  0.1617 | 0.5385 (0.3258-0.7512); 0.0000; 0.2900 | 0.4257 (0.1911-0.6603); 0.0003; 0.1812 | 0.5178 (0.2973-0.7383); 0.0000; 0.2681 | 0.3536 (0.1149-0.5922); 0.0022; 0.1250 | 0.4788 (0.2454-0.7121); 0.0001;  0.2292 | 0.4384  (0.1983-  0.6786);  0.0003;  0.1853 | 0.4021 (0.1680-0.6361); 0.1617;  0.0005 | 0.5599 (0.3561-0.7636); 0.3134;  0.0000 | 0.5429 (0.3236-0.7622); 0.2947;  0.0000 | 0.4304 (0.1903–0.6706); 0.1853;  0.0003 |
|  | Left | 0.3630  (0.1374-0.5586); 0.0012;  0.1318 | 0.4973  (0.2776-  0.7170);  0.0000;  0.2473 | 0.4984 (0.2751-0.7057); 0.0000; 0.2405 | 0.5242 (0.3068-0.7415); 0.0000; 0.2748 | 0.4722 (0.2513-0.6932); 0.0001; 0.2230 | 0.5935 (0.3878-0.7992); 0.0000; 0.3523 | 0.4383 (0.2052-0.6685); 0.0002;  0.1921 | 0.4683  (0.2409-  0.6957);  0.0001;  0.2193 | 0.4973 (0.2776-0.7170); 0.2473;  0.0000 | 0.3861 (0.1655-0.6066); 0.1490;  0.0005 | 0.4973 (0.2776-0.7170); 0.2473;  0.0000 | 0.4683 (0.2489–0.6957); 0.2153;  0.0001 |
| Wrist in the intermediate position, Y/N | Right | 0.4928  (0.2814-0.7042); 0.0001;  0.2428 | 0.2618  (0.0095-  0.5131);  0.0346;  0.0634 | 0.4928 (0.2814-0.7042); 0.0001; 0.2428 | 0.3682 (0.1841-0.6323); 0.0034; 0.1356 | 0.4321 (0.1900-0.6742); 0.0005; 0.1867 | 0.2666 (0.0010-0.5331); 0.0276; 0.0711 | 0.2998 (0.0217-0.5779); 0.0164;  0.0899 | 0.3187  (0.0342-  0.6032);  0.0122;  0.1016 | 0.2518 (0.0095-0.5031); 0.0634;  0.0300 | 0.4606 (0.2556-0.6662); 0.2124;  0.0001 | 0.5494 (0.3288-0.7701); 0.3019;  0.0000 | 0.3187 (0.0342–0.6032); 0.1016;  0.0111 |
|  | Left | 0.3467  (0.1051-0.5883); 0.0046;  0.1202 | 0.2869  (0.0227-  0.5511);  0.0196;  0.0823 | 0.4459 (0.2272-0.6646); 0.0003; 0.1988 | 0.4800 (0.1503-0.6695); 0.0014; 0.1680 | 0.3760 (0.1259-0.6262); 0.0027; 0.1414 | 0.3019 (0.0323-0.5761); 0.0155; 0.0911 | 0.3358 (0.0542-0.6174); 0.0090;  0.1128 | 0.3551  (0.0670-  0.6433);  0.0066;  0.1261 | 0.2869 (0.0227-0.5511); 0.0823;  0.0177 | 0.2248 (0.0172-0.4669); 0.0505;  0.0441 | 0.3923 (0.1376-0.6471); 0.1539;  0.0019 | 0.3551 (0.0670–0.6433); 0.1261;  0.0062 |
| Thumb outside, Y/N | Right | 0.4928  (0.2814-0.7842); 0.0001;  0.2428 | 0.2518  (0.0095-  0.5131);  0.0346;  0.0634 | 0.4928 (0.2814-0.7842); 0.0001; 0.2428 | 0.3682 (0.1041-0.6323); 0.0034; 0.1356 | 0.4321 (0.1900-0.6742); 0.0005; 0.1867 | 0.2666 (0.0010-0.5331); 0.0276; 0.0711 | 0.2998 (0.0217-0.5779); 0.0164;  0.0899 | 0.3187  (0.0342-  0.6032);  0.0122;  0.1016 | 0.2518 (0.0095-0.5031); 0.0634;  0.0300 | 0.5494 (0.3288-0.7701); 0.2124;  0.0000 | 0.3187 (0.0342-0.6032); 0.3019;  0.0111 | 0.3158 (0.1423–0.4892); 0.1016;  0.0038 |
|  | Left | 0.3467  (0.1051-0.5883); 0.0046;  0.1202 | 0.2869  (0.0227-  0.5511);  0.0196;  0.0823 | 0.4459 (0.2272-0.6646); 0.0003; 0.1988 | 0.4099 (0.1503-0.6695); 0.0014; 0.1680 | 0.3760 (0.1259-0.6262); 0.0027; 0.1414 | 0.3019 (0.0323-0.5715); 0.0155; 0.0911 | 0.3358 (0.0542-0.6174); 0.0090;  0.1128 | 0.3551  (0.0670-  0.6433);  0.0066;  0.1261 | 0.2869 (0.0227-0.5511); 0.0823;  0.0177 | 0.2248 (0.0172-0.4669); 0.0505;  0.0441 | 0.3923 (0.1376-0.6471); 0.1539;  0.0019 | 0.3551 (0.0670–0.6433); 0.1261;  0.0062 |
| Palm in the intermediate position, Y/N | Right | 0.4928  (0.2814-0.7842); 0.0001;  0.2428 | 0.2518  (0.0095-  0.5131);  0.0346;  0.0634 | 0.4928 (0.2814-0.7842); 0.0001; 0.2428 | 0.3682 (0.1041-0.6323); 0.0034; 0.1356 | 0.4321 (0.1900-0.6742); 0.0005; 0.1867 | 0.2666 (0.0.0010-0.5331); 0.0276; 0.0711 | 0.2998 (0.0217-0.5779); 0.0164;  0.0899 | 0.3187  (0.0342-  0.6032);  0.0122;  0.1016 | 0.2518 (0.0095-0.5031); 0.0634;  0.0300 | 0.4609 (0.2556-0.6662); 0.2124;  0.0001 | 0.5494 (0.3288-0.7701); 0.3019;  0.0000 | 0.3187 (0.0342–0.6032); 0.1016;  0.0111 |
|  | Left | 0.3467  (0.1050-0.5883); 0.0046;  0.1202 | 0.2869  (0.0227-  0.5511);  0.0196;  0.0823 | 0.4459 (0.2272-0.6646); 0.0003; 0.1988 | 0.4099 (0.1503-0.6695); 0.0014; 0.1680 | 0.3760 (0.1259-0.6262); 0.0027; 0.1414 | 0.3019 (0.0323-0.5715); 0.0155; 0.0911 | 0.3358 (0.0542-0.6174); 0.0070;  0.1128 | 0.3551  (0.0670-  0.6433);  0.0066;  0.1261 | 0.2869 (0.0227-0.5511); 0.0823;  0.0177 | 0.2248 (0.0172-0.4669); 0.0823;  0.0441 | 0.3923 (0.1376-0.6471); 01539;  0.0019 | 0.3551 (0.0670–0.6433); 01261  0.0062 |
| Pelvis extended (no anteversion or retroversion), Y/N | - | 0.4058  (0.1847-0.6270); 0.0003;  0.1647 | 0.5430  (0.3328-  0.7533);  0.0000;  0.2949 | 0.5936 (0.3950-0.7922); 0.0000; 0.3524 | 0.5034 (0.2865-0.7202); 0.0000; 0.2534 | 0.5816 (0.3786-0.7847); 0.0000; 0.3383 | 0.5034 (0.2865-0.7202); 0.0000; 0.2534 | 0.4918 (0.2723-0.7113); 0.0000;  0.2419 | 0.4498  (0.2242-  0.6753);  0.0001;  0.2023 | 0.4100 (0.1844-0.6356); 0.1681;  0.0003 | 0.4861 (0.2758-0.6963); 0.2363;  0.0000 | 0.4765 (0.2573-0.6957); 0.2271;  0.0000 | 0.4498 (0.2481–0.6515); 0.2023;  0.0000 |
| Lower limb situated in moderate external rotation, Y/N | Right | 0.4483  (0.2207-0.6759); 0.0002;  0.1679 | 0.4098  (0.1572-  0.6624);  0.0010;  0.2882 | 0.5368 (0.3324-0.7412); 0.0000; 0.2760 | 0.5263 (0.2855-0.7652); 0.0000; 0.2343 | 0.4840 (0.2502-0.7179); 0.0001; 0.1840 | 0.4290 (0.1725-0.6855); 0.0006; 0.2232 | 0. 4724 (0.2081-0.7368); 0.0003;  0.2474 | 0.4976  (0.2294-  0.7653);  0.0002;  0.1679 | 0.4098 (0.1572-0.6624); 0.1679;  0.0009 | 0.4169 (0.1953-0.6384); 0.1738;  0.0004 | 0.5039 (0.2670-0.7408); 0.2539;  0.0001 | 0.4973 (0.2776–0.7170); 0.2474;  0.0000 |
|  | Left | 0.4097  (0.1742-0.6452); 0.0006;  0.1679 | 0.4645  (0.2198-  0.7092);  0.0002;  0.2157 | 0.4942 (0.2737-0.7146); 0.0000; 0.2442 | 0.4856 (0.2379-0.7332); 0.0001; 0.2358 | 0.4450 (0.2033-0.6866); 0.0003; 0.1980 | 0.4856 (0.2379-0.7332); 0.0001; 0.2358 | 0.4363 (0.1702-0.7024); 0.0006;  0.1903 | 0.4607  (0.1909-  0.7384);  0.0004;  0.2122 | 0.3747 (0.1202-0.6291); 0.1404;  0.0020 | 0.2969 (0.0599-0.5338); 0.0881;  0.0090 | 0.4645 (0.2198-0.7092); 0.2157;  0.0002 | 0.3686 (0.0845–0.6366); 0.1300;  0.0038 |
| Lower limb bent at a right angle at hip and knee joints, foot in intermediate position; lifting above the substrate, Y/N | Right | 0.4231  (0.1896-0.6566); 0.0004;  0.1790 | 0.3159  (0.0639-  0.5678);  0.0074;  0.0998 | 0.5011 (0.2789-0.7234); 0.0000; 0.2511 | 0.4194 (0.1677-0.6712); 0.0006; 0.1759 | 0.4609 (0.2227-0.6992); 0.0001; 0.2125 | 0.3345 (0.0788-0.5901); 0.0051; 0.1119 | 0.3761 (0.1131-0.6392); 0.0022;  0.1415 | 0.3998  (0.1332-  0.6664);  0.0013;  0.1599 | 0.3159 (0.0639-0.5678); 0.0998;  0.0065 | 0.3896 (0.1609-0.6182); 0.1518;  0.0007 | 0.4819 (0.2415-0.7223); 0.2322;  0.0001 | 0.3998 (0.1332–0.6664); 0.1599;  0.0012 |
|  | Left | 0.4097  (0.1742-0.6452); 0.0006;  0.1679 | 0.3747  (0.1202-  0.6291);  0.0021;  0.1404 | 0.4942 (0.2737-0.7146); 0.0000; 0.2442 | 0.4856 (0.2379-0.7332); 0.0001; 0.2358 | 0.4450 (0.2033-0.6866); 0.0003; 0.1980 | 0.3936 (0.1362-0.6519); 0.0014; 0.1549 | 0.4363 (0.1702-0.7024); 0.0006;  0.1903 | 0.4607  (0.1909-  0.7384);  0.0004;  0.2122 | 0.3747 (0.1202-0.6291); 0.1404;  0.0020 | 0.2969 (0.0599-0.5338); 0.0881;  0.0090 | 0.4645 (0.2198-0.7092); 0.2157;  0.0002 | 0.3686 (0.0845–0.6366); 0.1300;  0.0038 |
