# Supplementary material for: Motor elements of the third month variously predict individual later motor elements
Source: Front Hum Neurosci. 2025 Jul 22;19:1586228. doi: 10.3389/fnhum.2025.1586228 (PMC12322975; doi:10.3389/fnhum.2025.1586228)
Supplement: Supplementary file 1 [file Supplementary_file_1.zip › Supplementary Material/Table_3.DOCX]

Table III. The impact of qualitative elements assessed at three months of age in the prone position on qualitative elements assessed at the age of 7–8 months. For each pair of variables, the values of Cramer’s V coefficient, confidence intervals, and Goodman and Kruskal Tau coefficients are given, along with exact p-value. The relatively strong correlations are marked with yellow.

| Qualitative characteristics in the prone position | Side of the body | Load on the hip and buttocks - R | Load on the hip and buttocks - L | Triangle support - R | Triangle support - L | Support on the upper limb straight, palm open- - R | Support on the upper limb straight, palm open- - L | Upper limb elevated to 120 degrees - R | Upper limb elevated to 120 degrees - L |
| --- | --- | --- | --- | --- | --- | --- | --- | --- | --- |
| Isolated head rotation, Y/N | - | 0.4322 (0.2415–0.6228); 0.1868;  0.0000 | 0.3999 (0.2046–0.5951); 0.1599;  0.0001 | 0.3548 (0.149–0.5606); 0.1259;  0.0000 | 0.3215 (0.1130–0.5301); 0. 1034;  0.0021 | 0.2348 (0.0140–0.4555); 0.0551;  0.0221 | 0.2773 (0.0541–0.4926); 0.0747;  0.0096 | 0.2878 (0.0769–0.4986); 0.0828;  0.0052 | 0.4185 (0.2256–0.6114); 0.1494;  0.0001 |
| Arm in front, forearm in the intermediate position, elbow outside of the line of the shoulder, Y/N | Right | 0.2988 (0.0968–0.5008); 0.0093;  0.0037 | 0.2604 (0.0547–0.4661); 0.0678;  0.0096 | 0.4069 (0.2012–0.6125); 0.1655;  0.0002 | 0.3686 (0.1503–0.5798); 0.1359;  0.0006 | 0.3062 (0.0753–0.5372); 0.0938;  0.0054 | 0.2826 (0.0526–0.5126); 0.0799;  0.0009 | 0.3298 (0.1156–0.5440); 0.1088;  0.0020 | 0.4254 (0.2309–0.6199); 0.1480;  0.0002 |
|  | Left | 0.2235 (0.0176–0.4293); 0.0499;  0.0206 | 0.2376 (0.01311–0.4441); 0.0565;  0.0156 | 0.3301 (0.1197–0.5484); 0.1009;  0.0018 | 0.3471 (0.1363–0.5580); 0.1205;  0.0011 | 0.2230 (0.0057–0.4516); 0.0497;  0.0296 | 0.2670 (0.0391–0.4948); 0.0713;  0.0122 | 0.3093 (0.0952–0.5234); 0.0957;  0.0033 | 0.4834 (0.2083–0.5986); 0.1318  0.0003 |
| Palm loosely open, Y/N | Right | 0.3158 (0.1423–0.4892); 0.0997;  0.0038 | 0.3245 (0.1491–0.4999); 0.1053;  0.0031 | 0.3838 (0.1952–0.5725); 0.1473;  0.0000 | 0.3952 (0.2040–0.5864); 0.1562;  0.0006 | 0.5953 (0.3692–0.8214); 0.3544;  0.0000 | 0.4698 (0.2198–0.7198); 0.2207;  0.0002 | 0.4070 (0.2132–0.6009); 0.1657;  0.0004 | 0.3109 (0.0983–0.5235); 0.0910;  0.0060 |
|  | Left | 0.1149 (0.0955–0.3253); 0.0132;  0.1705 | 0.1219 (0.0899–0.3338); 0.0149;  0.1591 | 0.2570 (0.0415–0.4725); 0.0660;  0.0216 | 0.2665 (0.0483–0.4866); 0.0710;  0.0183 | 0.3181 (0.0427–0.5936); 0.1012;  0.0110 | 0.5179 (0.2839–0.7519); 0.2682;  0.0001 | 0.1855 (0.0435–0.4146); 0.0344;  0.0756 | 0.2667 (0.0493–0.4821); 0.0668;  0.0177 |
| Thumb outside, Y/N | Right | 0.3158 (0.1423–0.4892); 0.0997;  0.0038 | 0.3245 (0.1491–0.4999); 0.1053;  0.0031 | 0.3838 (0.1952–0.5725); 0.1473;  0.0000 | 0.3952 (0.2040–0.5864); 0.1562;  0.0006 | 0.5953 (0.3692–0.8214); 0.3544;  0.0000 | 0.4698 (0.2198–0.7198); 0.2207;  0.0002 | 0.4070 (0.2132–0.6009); 0.1657;  0.0004 | 0.3109 (0.0983–0.5235); 0.0910;  0.0060 |
|  | Left | 0.1149 (0.0955–0.3253); 0.0132;  0.1705 | 0.1219 (0.0899–0.3338); 0.0149;  0.1591 | 0.2570 (0.0415–0.4725); 0.0660;  0.0216 | 0.2665 (0.0483–0.4866); 0.0710.  0.0183 | 0.3181 (0.0427–0.5936). 0.1012;  0.0110 | 0.5179 (0.2839–0.7519); 0.2682;  0.0001 | 0.1855 (0.0435–0.4146); 0.0344;  0.0756 | 0.2667 (0.0493–0.4821); 0.0668;  0.0177 |
| Spine segmentally in extension, Y/N | – | 0.3874 (0.1926–0.5682); 0.1501;  0.0002 | 0.3566 (0.1582–0.5549); 0.1271;  0.0006 | 0.3678 (0.1648–0.5789); 0.1363;  0.0005 | 0.3365 (0.1307–0.5423); 0.1132;  0.0013 | 0.3359 (0.1252–0.5467); 0.1128;  0.0020 | 0.2467 (0.0306–04627); 0.0608  0.0163 | 0.3565 (0.1512–0.5618); 0.1271;  0.0007 | 0.3806 (0.1877–0.5734); 0.1219;  0.0003 |
| Scapula situated in the medial position, Y/N | Right | 0.2670 (0.0630–0.4709); 0.0713;  0.0087 | 0.2245 (0.0167–0.4322); 0.0584;  0.0221 | 0.3069 (0.0929–0.5210); 0.0942;  0.0038 | 0.2634 (0.0461–0.4808); 0.0694;  0.0109 | 0.2854 (0.0464–0.5243); 0.0814;  0.0097 | 0.1930 (0.0405–0.4264); 0.0372;  0.0512 | 0.2786 (0.0599–0.4973); 0.0776;  0.0078 | 0.3457 (0.1467–0.5447); 0.0810  0.0015 |
|  | Left | 0.1943 (0.0126–0.4012); 0.0378;  0.0360 | 0.2079 (0.0002–0.4155); 0.0432;  0.0285 | 0.2414 (0.0274–0.4554); 0.0583;  0.0159 | 0.2571 (0.0421–0.4722); 0.0661;  0.0116 | 0.1687 (0.0592–0.3965); 0.0284;  0.0711 | 0.2156 (0.0135–0.4446); 0.0465;  0.0334 | 0.2170 (0.0005–0.4336); 0.0471;  0.0263 | 0.3288 (0.1327–0.5249); 0.0761;  0.0019 |
| Pelvis in the intermediate position, Y/N | – | 0.3510 (0.1533–0.5487); 0.1232;  0.0000 | 0.3130 (0.1104–0.5155); 0.0980;  0.0026 | 0.4620 (0.2619–0.6622); 0.2135;  0.0000 | 0.4244 (0.2184–0.6303); 0.1801;  0.0001 | 0.3062 (0.0753–0.5372); 0.0938;  0.0054 | 0.3862 (0.1753–0.5970); 0.1222;  0.0004 | 0.5280 (0.3424–07137); 0.1491;  0.0000 | 0.5421 (0.3452–0.7391); 0.2439;  0.0000 |
| Lower limbs situated loosely on the substrate, Y/N | Right | 0.2169 (0.1018–0.4919); 0.0881;  0.0052 | 0.3072 (0.1108–0.5037); 0.0944;  0.0041 | 0.3767 (0.1703–0.5831); 0.1419;  0.0007 | 0.3898 (0.1815–0.5981); 0.1519;  0.0005 | 0.2673 (0.0091–0.5254); 0.0714;  0.0190 | 0.2495 (0.0042–0.5031); 0.0622;  0.0252 | 0.4835 (0.1932–0.6137); 0.1628;  0.0004 | 0.3900 (0.1823–0.5978); 0.1432;  0.0005 |
|  | Left | 0.1989 (0.0083–0.4061); 0.0396;  0.0415 | 0.2091 (0.0006–0.4176); 0.0437;  0.0350 | 0.3438 (0.1318–0.5559); 0.1182;  0.0018 | 0.3570 (0.1431–0.5709); 0.1274;  0.0013 | 0.1605 (0.0840–0.4050); 0.0257;  0.0959 | 0.2270 (0.0223–0.4764); 0.0515;  0.0354 | 0.3016 (0.0797–0.5236); 0.0910;  0.0058 | 0.4556 (0.2619–0.6494); 0.1465;  0.0001 |
| Foot in the intermediate position, Y/N | Right | 0.1990 (0.0020–0.4000); 0.0396;  0.0566 | 0.2066 (0.0039–0.4093); 0.0427; 0.0503 | 0.2570 (0.0415–0.4725); 0.0660;  0.0216 | 0.2665 (0.0483–0.4866); 0.0710;  0.0183 | 0.4290 (0.1644–0.6936); 0.1840;  0.0010 | 0.4099 (01503–0.6695); 0.1680;  0.0013 | 0.2764 (0.05544–0.4973); 0.0764;  0.0154 | 0.2667 (0.0493–0.4842); 0.0668;  0.0177 |
|  | Left | 0.1149 (0.0955–0.3253); 0.0132;  0.1705 | 0.1219 (0.0899–0.3338); 0.0149;  0.1591 | 0.2570 (0.0415–0.4725); 0.0660;  0.0216 | 0.2665 (0.0483–0.4866); 0.0710;  0.0183 | 0.3181 (0.0427–0.5936); 0.1012;  0.0110 | 0.4099 (01503–0.6695); 0.1680;  0.0013 | 0.1855 (0.0435–0.4146); 0.0344;  0.0756 | 0.2667 (0.0493–0.4821); 0.0668;  0.0177 |
