# Supplementary material for: Motor elements of the third month variously predict individual later motor elements
Source: Front Hum Neurosci. 2025 Jul 22;19:1586228. doi: 10.3389/fnhum.2025.1586228 (PMC12322975; doi:10.3389/fnhum.2025.1586228)
Supplement: Supplementary file 1 [file Supplementary_file_1.zip › Supplementary Material/Table_4.DOCX]

Table IV. The impact of qualitative elements assessed at three months of age in the supine position on qualitative elements assessed at the age of 7–8 months. For each pair of variables, the values of Cramer’s V coefficient, confidence intervals, and Goodman and Kruskal Tau coefficients are given, along with exact p-value. The relatively strong correlations are marked with yellow.

| Qualitative characteristics in the supine position | Side of the body | Load on the hip and buttocks - R | Load on the hip and buttocks - L | Triangle support - R | Triangle support - L | Support on the upper limb straight, palm open - R | Support on the upper limb straight, palm open - L | Upper limb elevated to 120 degrees - R | Upper limb elevated to 120 degrees - L |
| --- | --- | --- | --- | --- | --- | --- | --- | --- | --- |
| Head  symmetry Y/N | - | 0.3497 (0.1526–0.5467); 0.1223;  0.0009 | 0.3091 (0.1064–0.5118); 0.0955;  0.0030 | 0.3411 (0.1294–0.5527); 0.1163;  0.0014 | 0.2994 (0.0839–0.5150); 0.0897;  0.0045 | 0.2686 (0.0323–0.5048); 0.0721;  0.0133 | 0.3161 (0.0818–0.5505); 0.0999;  0.0044 | 0.2571 (0.0389–0.4753); 0.0661;  0.0124 | 0.4213 (0.2260–0.6165); 0.1406;  0.0001 |
| Spine in  extension, Y/N | - | 0.4094 (0.2165–0.6024); 0.1676;  0.0001 | 0.3479 (0.1809–0.5748); 0.1428;  0.0003 | 0.4379 (0.2395–0.6363); 0.1918;  0.0000 | 0.4065 (0.2044–0.6085); 0.1652;  0.0001 | 0.3495 (0.1373–0.5617); 0.1221;  0.0014 | 0.3219 (0.1074–0.5364); 0.1036;  0.0028 | 0.3746 (0.1693–0.5799); 0.1403;  0.0004 | 0.4472 (0.2563–0.6380); 0.1748;  0.0000 |
| Shoulder in balance between external and internal rotation, Y/N | Right | 0.3498 (0.1541–0.5454); 0.1223;  0.0009 | 0.3063 (0.1038–0.5088); 0.0938;  0.0034 | 0.4498 (0.2481–0.6515); 0.2023;  0.0000 | 0.4062 (0.1968–0.6156); 0.1650;  0.0002 | 0.3772 (0.1372–0.6171); 0.1422;  0.0011 | 0.2815 (0.0421–0.5208); 0.0792;  0.0105 | 0.4226 (0.2122–0.6329); 0.1786;  0.0001 | 0.4720 (0.2813–0.6628); 0.1804;  0.0000 |
|  | Left | 0.2957 (0.0937–0.4977); 0.0874;  0.0042 | 0.3091 (0.1064–0.5118); 0.0955;  0.0030 | 0.3981 (0.1908–0.6054); 0.1585;  0.0002 | 0.4148 (0.2068–0.6227); 0.1720;  0.0001 | 0.2686 (0.0323–0.5048); 0.0721;  0.0133 | 0.3161 (0.0818–0.5505); 0.0999;  0.0044 | 0.3737 (0.1603–0.5871); 0.1397;  0.0006 | 0.4730 (0.2818–0.6641); 0.1861;  0.0000 |
| Wrist in the intermediate position, Y/N | Right | 0.3158 (0.1423–0.4892); 0.0132;  0.0038 | 0.3245 (0.1491–0.4999); 0.0149;  0.0031 | 0.3838 (0.1952–0.5725); 0.0660;  0.0000 | 0.3952 (0.2040–0.5864); 0.0710;  0.0006 | 0.5953 (0.3692–0.8241); 0.1012;  0.0000 | 0.4698 (0.2198–0.7198); 0.2682;  0.0002 | 0.4070 (0.2132–0.6009); 0.0344;  0.0004 | 0.3109 (0.0983–0.5235); 0.0668;  0.0060 |
|  | Left | 0.1149 (0.0955–0.3253); 0.0997;  0.1705 | 0.1219 (0.0899–0.3338); 0.1053;  0.1591 | 0.2570 (0.04115–0.4725); 0.1473;  0.0216 | 0.2570 (0.04115–0.4725); 0.1562;  0.0216 | 0.3161 (0.0427–0.5936); 0.3544;  0.0110 | 0.5179 (0.2839–0.7519); 0.2207;  0.0001 | 0.1855 (0.0425–0.4146); 0.1657;  0.0756 | 0.2667 (0.0493–0.4842); 0.0910;  0.0177 |
| Thumb  outside, Y/N | Right | 0.3158 (0.1423–0.4892); 0.0132;  0.0038 | 0.3245 (0.1491–0.4999); 0.0149;  0.0031 | 0.3838 (0.1952–0.5725); 0.0660;  0.0000 | 0.3952 (0.2040–0.5864); 0.0710;  0.0006 | 0.5953 (0.3692–0.8241); 0.1012;  0.0000 | 0.4698 (0.2198–0.7198); 0.2682;  0.0002 | 0.4070 (0.2132–0.6009); 0.0344;  0.0004 | 0.3109 (0.0983–0.5235); 0.0608;  0.0060 |
|  | Left | 0.1149 (0.0955–0.3253); 0.1223;  0.1705 | 0.1219 (0.0899–0.3338); 0.0955;  0.1591 | 0.2570 (0.04115–0.4725); 0.1163;  0.0216 | 0.2665 (0.0483–0.4866); 0.0897;  0.0183 | 0.3181 (0.0427–0.5936); 0.0721;  0.0110 | 0.5179 (0.2839–0.7519); 0.0999;  0.0001 | 0.1855 (0.0435–0.4146); 0.0661;  0.0753 | 0.2667 (0.0493–0.4842); 0.1406;  0.0177 |

| Palm in the intermediate position, Y/N | Right | 0.3158 (0.1423–0.4892); 0.0997;  0.0038 | 0.3245 (0.1491–0.4999); 0.1053;  0.0031 | 0.3838 (0.1952–0.5725); 0.1473;  0.0000 | 0.3952 (0.2040–0.5864); 0.1562;  0.0006 | 0.5953 (0.3692–0.8241); 0.3544;  0.0000 | 0.4698 (0.2198–0.7198); 0.2207;  0.0002 | 0.4070 (0.2132–0.6009); 0.1657;  0.0004 | 0.3109 (0.0983–0.5235); 0.0910;  0.0060 |
| --- | --- | --- | --- | --- | --- | --- | --- | --- | --- |
|  | Left | 0.1149 (0.0955–0.3253); 0.0132;  0.1705 | 0.1219 (0.0899–0.3338); 0.0149;  0.1591 | 0.2570 (0.04115–0.4725); 0.0660;  0.0216 | 0.2665 (0.0483–0.4866); 0.0710;  0.0183 | 0.3181 (0.0427–0.5936); 0.1012;  0.0110 | 0.5179 (0.2839–0.7519); 0.2682;  0.0001 | 0.1855 (0.0435–0.4146); 0.0344;  0.0753 | 0.2667 (0.0493–0.4842); 0.0668;  0.0177 |
| Pelvis extended (no anteversion or retroversion), Y/N | - | 0.3236 (0.1237–0.5236); 0.1047; 0.0019 | 0.2842 (0.0797–0.4887); 0.8080;  0.0056 | 0.4865 (0.2891–0.6839); 0.2367;  0.0000 | 0.4478 (0.2436–0.6520); 0.2005;  0.0000 | 0.3225 (0.0093–0.5558); 0.1040;  0.0030 | 0.3671 (0.1372–0.5969); 0.1347;  0.0011 | 0.4085 (0.1986–0.6185); 0.1669;  0.0002 | 0.5532 (0.3713–0.7351); 0.2687;  0.0000 |
| Lower limb situated in moderate external rotation, Y/N | Right | 0.2712 (0.0792–0.4633); 0.0736;  0.0116 | 0.2802 (0.0864–0.4739); 0.0785;  0.0096 | 0.3402 (0.1344–0.5459); 0.1157;  0.0026 | 0.3515 (0.1434–0.5596); 0.1236;  0.0020 | 0.3509 (0.0822–0.6196); 0.1231;  0.0045 | 0.3326 (0.0688–0.5965); 0.1106;  0.0061 | 0.3634 (0.1528–0.5739); 0.1320;  0.0015 | 0.3517 (0.1439–0.5594); 0.1166;  0.0019 |
|  | Left | 0.2319 (0.0302–0.4336); 0.0538;  0.0257 | 0.2410 (0.0378–0.4442); 0.0581;  0.0216 | 0.3775 (0.1764–0.5787); 0.1425;  0.0000 | 0.3897 (0.1864–0.5931); 0.1519;  0.0006 | 0.2253 (0.0361–0.4866); 0.0508;  0.0434 | 0.3016 (0.0403–0.5630); 0.0910;  0.0107 | 0.3252 (0.1062–0.5441); 0.1057;  0.0039 | 0.3899 (0.1868–0.5929); 0.1435;  0.0006 |
| Lower limb bent at a right angle at hip and knee joints, foot in intermediate position; lifting above the substrate, Y/N | Right | 0.2307 (0.0266–0.4348); 0.0532;  0.0240 | 0.2406 (0.0352–0.4461); 0.0579;  0.0198 | 0.3068 (0.0913–0.5222); 0.0941;  0.0051 | 0.3191 (0.1017–0.5366); 0.1018;  0.0038 | 0.3245 (0.1491–0.4999); 0.1256;  0.0031 | 0.2495 (0.0042–0.5031); 0.0672;  0.0252 | 0.3320 (0.1125–0.5515); 0.1102;  0.0029 | 0.3804 (0.1643–0.5966); 0.0780;  0.0014 |
|  | Left | 0.1602 (0.0486–0.3691); 0.0257;  0.0052 | 0.1689 (0.0413–0.3792); 0.0285;  0.0758 | 0.3019 (0.0875–0.5162); 0.0911;  0.0064 | 0.3133 (0.0967–0.5299); 0.0981;  0.0050 | 0.2253 (0.0361–0.4866); 0.0508;  0.0434 | 0.3016 (0.0403–0.5630); 0.0910;  0.0107 | 0.2478 (0.0220–0.4736); 0.0614;  0.0218 | 0.3137 (0.0978–0.5295); 0.0923  0.0048 |
